# The responses of subjective feeling, task performance ability, cortisol and HRV for the various types of floor impact sound: a pilot study

**DOI:** 10.1186/s40557-017-0168-x

**Published:** 2017-05-15

**Authors:** Seok Hyeon Yun, Sang Jin Park, Chang Sun Sim, Joo Hyun Sung, Ahra Kim, Jang Myeong Lee, Sang Hyun Lee, Jiho Lee

**Affiliations:** 1 Department of Occupational and Environmental Medicine, Ulsan University Hospital, University of Ulsan College of Medicine, 877, Bangeojinsunhwando-ro, Dong-gu, Ulsan, 44033 Republic of Korea; 2 Environmental Health Center, Ulsan University Hospital, Ulsan, Republic of Korea; 3 Department of Mechanical Engineering, University of Ulsan College of Engineering, Ulsan, Republic of Korea; 4 Ulsan Science High School, Ulsan, Republic of Korea

**Keywords:** Floor impact sound (FIS), Annoyance, English listening test, Cortisol, Heart rate variability (HRV)

## Abstract

**Background:**

Recently, noise coming from the neighborhood via floor wall has become a great social problem. The noise between the floors can be a cause of physical and psychological problems, and the different types of floor impact sound (FIS) may have the different effects on the human’s body and mind. The purpose of this study is to assess the responses of subjective feeling, task performance ability, cortisol and HRV for the various types of floor impact.

**Methods:**

Ten men and 5 women were enrolled in our study, and the English listening test was performed under the twelve different types of FIS, which were made by the combinations of bang machine (B), tapping machine (T), impact ball (I) and sound-proof mattress (M). The 15 subjects were exposed to each FIS for about 3 min, and the subjective annoyance, performance ability (English listening test), cortisol level of urine/saliva and heart rate variability (HRV) were examined. The sound pressure level (SPL) and frequency of FIS were analyzed. Repeated-measures ANOVA, paired t-test, Wilcoxon signed rank test were performed for data analysis.

**Results:**

The SPL of tapping machine (T) was reduced with the soundproof mattress (M) by 3.9–7.3 dBA. Impact ball (I) was higher than other FIS in low frequency (31.5–125 Hz) by 10 dBA, and tapping machine (T) was higher than other FIS in high frequency (2–4 k Hz) by 10 dBA. The subjective annoyance is highest in the combination of bang machine and tapping machine (BT), and next in the tapping machine (T). The English listening score was also lowest in the BT, and next in T. The difference of salivary cortisol levels between various types of FIS was significant (*p* = 0.003). The change of HRV parameters by the change of FIS types was significant in some parameters, which were total power (TP) (*p* = 0.004), low frequency (LF) (*p* = 0.002) and high frequency (HF) (*p* = 0.011).

**Conclusions:**

These results suggest that the human’s subjective and objective responses were different according to FIS types and those combinations.

## Background

Currently, most of the housing in Korea is communal, and floor impact sound (FIS) between levels is as an important societal issue. According to the 2014 Korean Housing Survey conducted by the Ministry of Land, Infrastructure and Transport, the proportion of communal housing exceeded one half of all housing units (59.2%), including 49.6% for apartments, 3.4% for townhouses and 6.2% for multi-family housing units [1]. In addition, the 2013 data available from the Korea Research Institute for Human Settlements showed that the rate of settling in a communal housing type including apartments reached as high as 89.9% of all new constructions; thus, the proportion of communal housing units is expected to increase further. Moreover, the number of consultation cases registered at the National Noise Information System operated by the Korea Environment Corporation under the Ministry of the Environment has continuously increased: 8795 cases in 2012, 18,524 in 2013, 20,641 in 2014, 19,278 in 2015 and finally, 2962 in January and February of 2016 [2].

Commonly, FIS is classified into three categories, light weight impact sounds (e.g., sounds created when a light object is dropped, when furniture is dragged, etc.), heavy weight impact sounds (e.g., sounds created when children are running), and airborne sounds (e.g., sounds from the TV). There were 15,776 cases registered at the field diagnostic service for FIS of the National Noise Information System over 49 months between March 2012 and March 2016. Of these, 11,474 (72.7%) were from heavy weight impact sounds created by children running or footsteps, 654 (4.1%) were related to noise due to hammering, 506 (3.2%) were from noise caused by furniture, and 466 (3.0%) were from noise caused by household appliances (TV, vacuum, washing machine, etc.). By housing type, cases were most frequently registered for apartments (12,690 cases; 80.4%), and residential buildings constructed before 1999 comprised 3500 cases (22.2%). Additionally, dwellings on a lower floor comprised 12,740 cases (80.8%) [2].

A multidisciplinary approach is necessary in addressing the societal issues involving FIS. To date, research has been mainly conducted in the engineering field, including the development of standard floor impact sources [3, 4] and a noise reduction effect achieved by floors of different thicknesses. However, impact on the human body should also be considered in establishing a policy to address an FIS-related issue or in developing building materials to reduce FIS. Until now, there have been rare investigations into the effect of FIS on human health from a medical point of view.

For the general noise, there is already a large scientific body of evidence on the effects of noise on annoyance [5–8], performance [9, 10]. Most of the studies on association noise and cortisol levels have been showed that cortisol levels were increased after noise exposure [11–17]. And noise exposure was associated with change in heart rate variability (HRV) [18–21].

For FIS, based on the effect of general noise on health, some studies have argued that FIS can cause problems such as hearing impairment, hindrance to conversation, decreased job performance, sleep disturbance, annoyance, fatigue, displeasure and elevated blood pressure [22], but the direct effect of FIS on health remains unclear. FIS is transmitted not only by air but also through a wall or a floor, and has a characteristic low frequency sound pattern [23]. Therefore, FIS can affect the human body differently than other types of noise. Although some research has been conducted concerning low frequency noise, this is not the case for FIS per se. There are various types of low frequency noise and we cannot exclude that different types of low frequency noise may have different effects on the human body.

Accordingly, in the present study we aimed to assess the responses of subjective feeling, task performance ability, cortisol and HRV for the various types of floor impact. To do so, we designed several experimental conditions involving FIS by using three standard floor impact sources, and measured study participants’ stress responses, including subjective feelings (displeasure and annoyance), task performance ability (English listening test), stress hormone levels (cortisol) and heart rate variability (HRV).

## Methods

### Subjects

Participants were recruited with an advertisement posted at a university between April and May 2014, and 15 participants (10 men and 5 women) were included in the study. Written consent forms and questionnaires were distributed to participants during a visit 2 weeks prior to the experiment, wherein they were instructed to limit smoking and coffee consumption until the experiment and to not perform a high intensity exercise nor drink alcohol from 2 days before the experiment. The signed written consent form and the completed survey were collected on the day of experiment. Those with a hearing problem or illness, or those taking medication were excluded. The study was approved by the Ulsan University Hospital Institutional Review Board (UUH 2014-08-008).

### Study design

#### Questionnaire

The questionnaire was designed to collect the following information: demographic characteristics (sex, age, drinking, smoking, coffee consumption, etc.), housing type, economic status, traffic patterns during commute hours and around their house, general health status, trauma and other health histories, awareness of environmental noise, noise sensitivity, hearing ability and an experience of tinnitus, hobbies and military service history. Additionally, the questionnaire included a psychosocial well-being scale, a resilience scale and the Korean Occupational Stress Scale. Based on the noise sensitivity score, participants were grouped into a low sensitivity group (score range: 0–3), a medium sensitivity group (4–6), and a high sensitivity group (7–10).

#### Floor impact sources

**Fig. 1:**
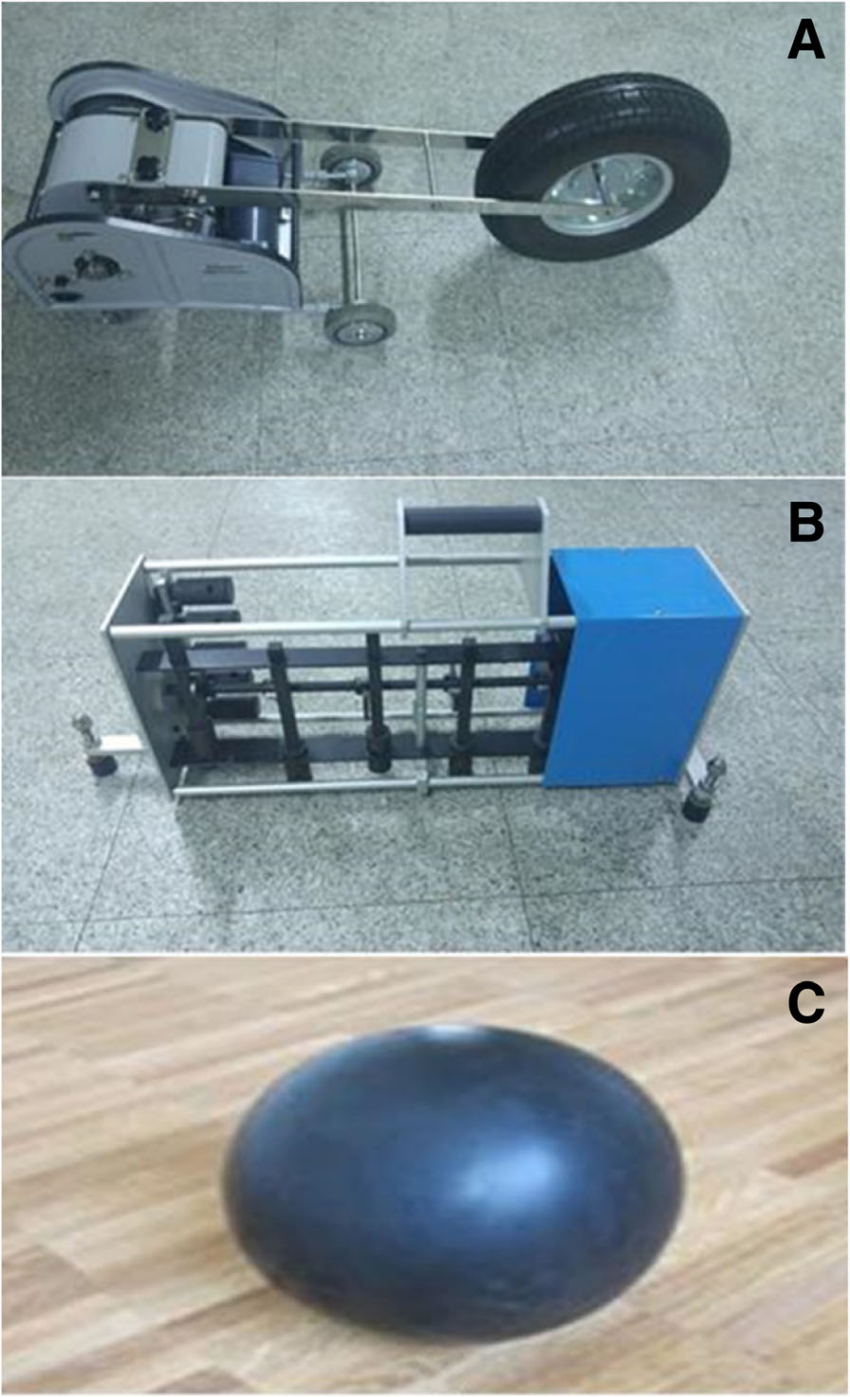
The sources of floor impact sound. **a** Bang machine (S&V Type T(FI-02)), **b** Tapping machine (S&V N-211), **c** Impact ball (self-made)

**Fig. 2:**
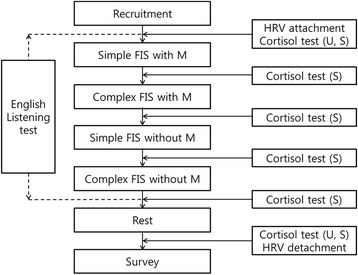
Flow chart of quasi-experimental process for evaluation on biological response of floor impact sound with or without soundproof mattress Abbreviation: FIS; floor impact sound, M; soundproof mattress, U; urine, S; saliva

The experiment was conducted in a university lecture room located on the 2^nd^ floor of a concrete building. The university building was built in 1972. The thickness of the floor is thicker and stronger than that of a typical home, because it is a structure that needs to be exposed to many loads of students. But, no buffer materials have been added to prevent floor impact sound, because the university building is not a residential area. From the point of view of the floor impact sound, it is expected that it will show the similar level of FIS as the apartments built in the 1990s. The floor impact sources were used on a 3^rd^ floor room immediately above where the experiment was conducted. Various FIS environments were created by utilizing three types of floor impact sources and a soundproof mattress. The three floor impact sources were a bang machine (S&V Type T [FI-02]), a tapping machine (S&V N-211), and an impact ball (manufactured specifically for the study following the standardization for impact balls, and weighing approximately 2.5 kg) (Figs. 1 and 2).

Regarding the bang machine, the impact weight, force and period were 7.0 kg, 387 N, and 1.7 ± 0.2 s, respectively. On the tapping machine, 5 hammers were attached to a line, each with a weight of 500 ± 5 g; the tapping frequency was 10 impacts/s, and the distance of a fall was 40 mm. The impact ball was manufactured specifically for the experiment, following the ISO standardization (ISO 140–11), such that an impact force of approximately 1500 N would be generated when the 2.5-kg rubber ball was free-falling from a height of 1 m. The bang machine is a standard heavy weight impact source used to generate heavy weight impact sounds such as that of children jumping or running; the tapping machine is a standard light weight impact source used to generate light weight impact sounds such as that of a light object being dropped or of a woman walking in high heels. The impact ball is also a standard heavy weight impact source, recently developed to overcome a limitation of the bang machine (i.e., overly strong stimulation in a low frequency band [below 63 Hz]) [4]. A total of 12 experimental conditions involving FIS were created in the following way: there were 3 types of floor impact sources: bang machine (B), tapping machine (T) and impact ball (I). These were either used alone (simple sound conditions: B, T and I) or in pairs (complex sound conditions: BT, TI and BI), both with or without a soundproof mattress (with M and without M). The sound exposure time was approximately 3 min for each FIS condition, and the total duration of the experiment was approximately 80 min.

#### Evaluation of the effect of FIS

##### Subjective perceptions

To assess subjective perceptions of FIS presented during the experiment, participants were asked 3 Questions immediately following the experiment; “which of the sounds was the most unpleasant sound?”, “which was the most disturbing sound?”, and “what is your annoyance score?”. The unpleasant sound means feeling bad or unwanted sound and the disturbing sound is relatively long term, worse than unpleasant sound and can affect physiologic condition. The average score of annoyance data were collected using an 11-point visual analog scale (VAS, with a score range of 0–10) based on ISO/TS 15666 (2003).

##### Task performance ability

Usually noise masks on listening ability and disturb concentration of work and performance, therefore English listening test in noise is proper method for performance ability influenced by adverse impact of noise. Participants were instructed to listen to spoken English and answer multiple choice questions where they were required to fill in the blanks with an appropriate word or sentence. The spoken English was sampled from the soundtrack used in an actual English listening test, and was presented at a comfortable volume (approximately 58 dBA). The questions were at a medium level of difficulty, with 1 min allocated per question. Participants were presented with two questions in each FIS condition. The maximum score for each question was 7 points.

##### Salivary and urinary cortisol

Two types of specimens were collected (urine and saliva). Urinary cortisol was measured twice from the urine specimens collected before and after the experiment, and salivary cortisol was measured for a total of 6 specimens collected before and after the experiment (2 times) and at those points when the type of FIS largely changed (4 times). The urinary and salivary specimens were cryogenically preserved and then processed using the enzyme-linked immunosorbent assay method immediately after being received at the laboratory.

##### Heart rate variability (HRV)

A portable HRV measuring device (TRI00A, Taewoong Medical, Korea) was attached to the participant’s chest before the experiment began and detached when it was over. The HRV data were analyzed using the software provided by the manufacturer (T-REX program, Taewoong Medical, Korea). Specifically, time domain analysis and frequency domain analysis were performed. Based on the time domain analysis, SDNN (standard deviation of normal to normal) and RMSSD (the square root of the mean of the sum of the square of differences between adjacent NN intervals) were obtained; based on the frequency domain analysis, TP (total power), LF (low frequency), HF (high frequency), LF/HF ratio, nLF (normalized low frequency) and nHF (normalized high frequency) were obtained.

### Statistical analysis

The English test scores across the FIS types were analyzed with repeated measures ANOVA (analysis of variance) and Wilcoxon signed ranks tests, and the urinary cortisol levels before and after exposure to FIS were analyzed using paired t-tests. Additionally, the salivary cortisol levels and changes in HRV across the FIS types were analyzed with repeated measures ANOVA. The statistical significance was determined when a *p* value was lower than α-error (0.05). All statistical analyses were performed using SPSS 21.0 (IBM SPSS Inc., Chicago, IL).

## Results

### General characteristics of subjects

The general characteristics of subjects were showed in Table 1. The average age of participants was 23.87 ± 1.68 years, and there were 10 men and 5 women. No one had a notable medical history or hearing impairment. Two participants were smokers, and 8 drank coffee on a daily basis. Eleven participants were alcohol drinkers. Regarding noise sensitivity, three participants were classified into the low sensitivity group (sensitivity score 0–3), 8 were classified into the medium sensitivity group (sensitivity score 4–6), and 4 were classified into the high sensitivity group (sensitivity score 7–10).

**Table 1:** General characteristics of subjects

Variable	N (Mean $\pm$ SD or %)
Age (years)	15 (23.87 $\pm$ 1.68)
Male	10 (66.67%)
Smoking	
Non smoker	13 (86.87%)
Current smoker	2 (13.33%)
Coffee consumption	
No	7 (46.67%)
Once a day	5 (33.33%)
Twice a day	3 (20%)
Drinking alcohol	
No	4 (26.67%)
Once or twice a week	3 (20%)
Once or twice a month	8 (53.33%)
Current medication	0 (0%)
Hearing impairment	0 (0%)
Noise sensitivity	
Low	3 (20%)
Middle	8 (53.33%)
High	4 (26.67%)

### Sound pressure level (SPL) and frequency analysis of FIS used in the experiment

**Fig. 3:**
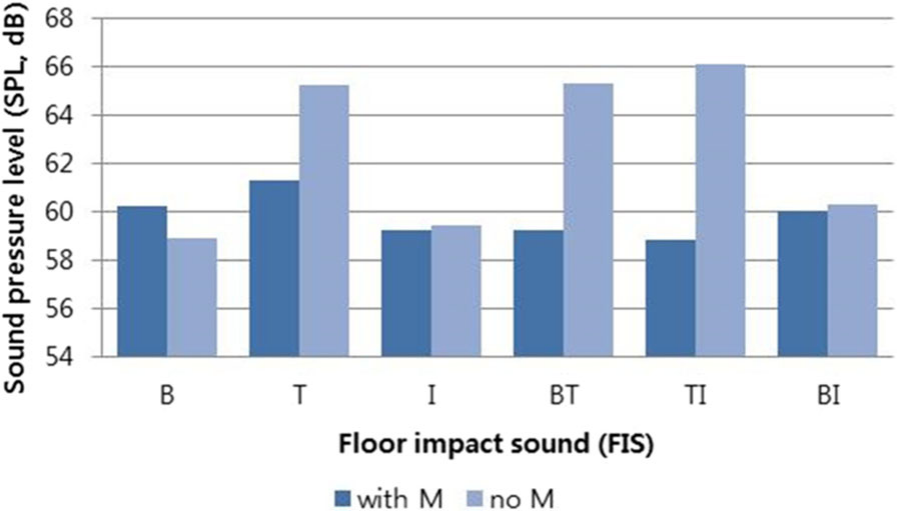
Sound pressure level (SPL) of floor impact sounds (FIS) with or without soundproof mattress. Abbreviation: B, bang machine; T, tapping machine; I, impact ball; BT, B and T; TI. T and I; BI, B and I; M, soundproof mattress

The SPL of background noise in the experimental room was 43.5 dBA, and the SPL of the English listening test alone was approximately 58 dBA. SPL of the FIS types was between 58.8 and 66.1 dBA. SPL of FIS was in the range of 58.8–61.3 dBA with a soundproof mattress, whereas it was in the range of 58.9–66.1 dBA without a soundproof mattress. Of the conditions wherein a soundproof mattress was not used, SPL was approximately 5 dBA higher when the tapping machine was used (i.e., T, BT and TI), with the highest SPL in TI (66.1 dBA). The examination of SPLs with and without a soundproof mattress showed that the difference was the greatest in TI (a difference of 7.3 dBA), followed by BT (a difference of 6.1 dBA) and T (a difference of 3.9 dBA) (Fig. 3).

**Fig. 4:**
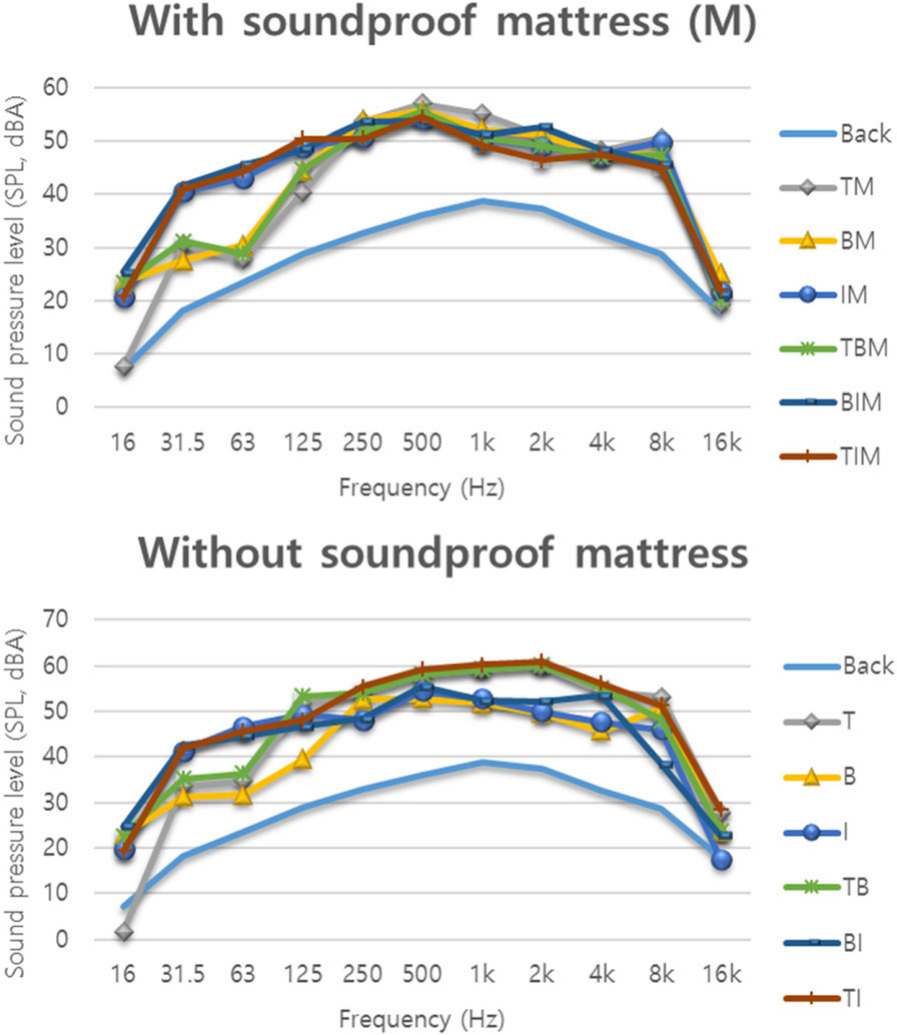
Frequency analysis of floor impact sounds (FIS) and their combinations. Abbreviation: Back, background; B, bang machine; T, tapping machine; I, impact ball; M, soundproof mattress; BT, B and T; TI, T and I; BI, B and I; BM, B with M; TM, T with M; IM, I with M; BTM, B and T with M; TIM, T and I with M; BIM, B and I with M

Frequency analysis on 12 FIS types showed that regardless of whether or not a soundproof mattress was used, SPL of a low frequency band (31.5–125 Hz) was higher in the conditions where the impact ball was used (i.e., I, TI and BI). Particularly, a large variation in SPL (approximately 10 dBA) was observed in those conditions with a soundproof mattress. In contrast, SPL was higher for a high frequency band (over 500 Hz) in the conditions where the tapping machine was used (i.e., T, BT and TI) in comparison to other FIS conditions, if a soundproof mattress was not used. Particularly, SPL was approximately 10 dBA higher in a high frequency band of 2000–4000 Hz (Fig. 4).

### Effect of FIS (subjective/objective responses)

#### Subjective responses

**Fig. 5:**
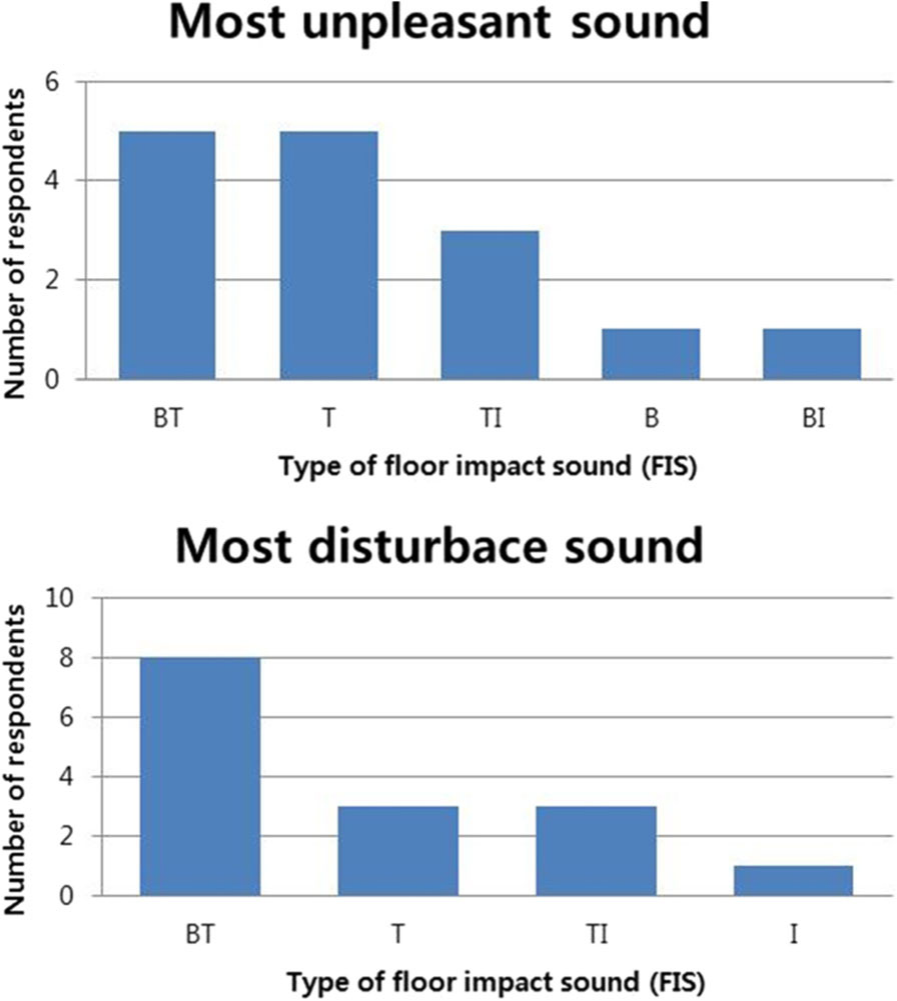
Subjective annoyance for the various types of floor impact sound (FIS). Abbreviation: B, bang machine; T, tapping machine; I, impact ball; BT, B and T; TI, T and I; BI, B and I

The average score of annoyance, measured on a 11-point VAS, was 7.27 ± 1.73. And for the most unpleasant sound, 5 participants chose BT, 5 chose T, 3 chose TI, 1 chose B and lastly, 1 chose BI. For the most disturbing sound, 8 participants chose BT, 3 chose T, another 3 chose TI and lastly, 1 chose I. Thus, generally, sound generated by sources including the tapping machine was perceived as unpleasant and disturbing (Fig. 5).

#### Task performance

Scores on the English listening test (the maximum possible score of 7 points per question) showed an overall significant difference across the FIS types (*p* < 0.001). More specifically, the overall difference was significant in the conditions without a soundproof mattress (*p* < 0.001), whereas no significant difference was found in the conditions with a soundproof mattress (*p* = 0.271). Of the conditions wherein a soundproof mattress was not used, the score was the lowest in BT (3.27 ± 1.67), followed by T (4.00 ± 2.20) and TI (5.20 ± 2.08) in ascending order.

Additionally, the scores for conditions with and without the use of a soundproof mattress were compared for each floor impact source. For B, T, BT, the score was significantly changed by using of soundproof mattress (*p* = 0.005, 0.007 and 0.004, respectively). The score was significantly decreased in the T, BT, although significantly increased in the B. (Table 2).

**Table 2:** English listening score with or without soundproof mattress (M) under various floor impact sound

	B	T	I	BT	TI	BI	*p*-value*
with mattress	5.53 ± 1.46	6.00 ± 1.36	6.40 ± 1.68	5.80 ± 1.93	6.00 ± 1.56	6.33 ± 1.29	0.271
without mattress	6.73 ± 0.59	4.00 ± 2.20	6.40 ± 1.30	3.27 ± 1.67	5.20 ± 2.08	6.60 ± 0.91	<0.001
*p*-value^†^	0.005	0.007	1.000	0.004	0.162	0.458	

Abbreviation: *B* bang machine, *T* tapping machine, *I* impact ball, *BT* B and T, *TI* T and I, *BI* B and I

Unit: mean ± standard deviation

*p-value was calculated by repeated measures ANOVA

^†^ *p*-value was calculated by Wilcoxon signed ranks test

#### Stress hormone (cortisol)

The changes of urinary cortisol level showed a decreasing trend (from 8950.8 ± 1837.4 pg/mL to 8914.5 ± 2044.9 pg/mL), but not significant (*p* = 0.889). The changes of salivary cortisol was significant (*p* = 0.003). The salivary cortisol level showed a decreasing trend, but the level increased at first exposure to FIS, and first exposure with and without mattress (Table 3). The comparison between simple and complex sounds conducted separately with and without a soundproof mattress showed that the salivary cortisol level significantly decreased when FIS changed from simple to complex sounds when a soundproof mattress was used (*p* = 0.002), whereas the decrease was not statistically significant when a soundproof mattress was not used (*p* = 0.074).

**Table 3:** The salivary and urinary cortisol level according to floor impact sounds (FIS)

	Pre exposure	S (M)	C (M)	S	C	Post exposure	*p*-value
salivary cortisol (pg/ml)	1582.6 ± 756.5	1700.3 ± 742.8	1357.9 ± 575.7	1373.4 ± 475.6	1237± 384.2	1089.1 ± 344.6	0.003*
urinary cortisol (pg/ml)	8950.8 ± 1837.4	-	-	-	-	8914.5 ± 2044.9	0.889^†^

Abbreviation: *S* simple sound, *C* complex sound, *M* soundproof mattress, *S (M)* S with M, *C (M)* C with M

Unit: mean ± standard deviation

*p-value was calculated by repeated measures ANOVA

^†^ *p*-value was calculated by paired t-test

#### HRV

Some HRV indices showed statistically significant differences depending on the FIS type: SDNN (*p* = 0.015), TP (*p* = 0.004), LF (*p* = 0.002), and HF (*p* = 0.011). HRV indexes were examined separately with and without a soundproof mattress, and HR (*p* = 0.025), SDNN (*p* = 0.013), TP (*p* = 0.014), LF (*p* = 0.030), and HF (*p* = 0.013) showed a significant difference in conditions with a soundproof mattress, whereas only HR (*p* < 0.001) showed a significant difference in conditions without a soundproof mattress (Table 4).

**Table 4:** Heart rate variability (HRV) parameters with or without soundproof mattress (M) under various floor impact sound

		B	T	I	BT	TI	BI	*p*-value*
HR	With mattress	84.05 ± 8.25	81.10 ± 7.20	83.58 ± 6.78	81.04 ± 7.30	81.92 ± 7.52	82.22 ± 6.89	0.025
	Without mattress	81.28 ± 1.93	81.65 ± 2.22	83.69 ± 1.79	80.41 ± 1.94	81.11 ± 1.95	82.86 ± 1.87	<0.001
SDNN	With mattress	20.66 ± 6.46	24.08 ± 7.06	20.85 ± 5.31	23.30 ± 6.44	21.94 ± 7.51	22.60 ± 6.42	0.013
	Without mattress	26.44 ± 16.01	22.84 ± 7.47	20.54 ± 6.21	22.73 ± 5.96	22.95 ± 7.64	20.48 ± 6.99	0.248
TP	With mattress	381.98 ± 263.46	559.24 ± 342.65	373.79 ± 156.62	473.85 ± 240.66	442.89 ± 283.62	485.09 ± 253.14	0.014
	Without mattress	849.46 ± 1307.7	507.60 ± 311.38	399.75 ± 246.66	462.79 ± 215.36	506.86 ± 335.45	382.88 ± 240.84	0.261
LF	With mattress	138.55 ± 104.63	213.46 ± 132.68	136.30 ± 56.39	170.19 ± 91.63	159.00 ± 101.34	187.27 ± 114.29	0.030
	Without mattress	382.20 ± 718.90	180.23 ± 113.28	147.89 ± 94.28	167.41 ± 66.61	199.74 ± 153.35	138.48 ± 88.66	0.274
HF	With mattress	243.43 ± 162.08	345.79 ± 213.76	237.48 ± 105.91	303.68 ± 161.93	283.85 ± 187.12	297.79 ± 157.14	0.013
	Without mattress	467.25 ± 595.76	327.36 ± 210.34	251.88 ± 156.28	295.35 ± 159.69	307.12 ± 193.18	244.40 ± 160.45	0.245
LF/HF	With mattress	0.51 ± 0.14	0.55 ± 0.11	0.52 ± 0.11	0.52 ± 0.13	0.52 ± 0.16	0.52 ± 0.15	0.900
	Without mattress	0.57 ± 0.24	0.54 ± 0.16	0.54 ± 0.09	0.54 ± 0.14s	0.54 ± 0.15	0.58 ± 0.13	0.744

Abbreviation: *B* bang machine, *T* tapping machine, *I* impact ball, *BT* B and T, *TI* T and I, *BI* B and I, *HR* heart rate, *SDNN* standard deviation normal to normal, *TP* total power, *LF* low frequency, *HF* high frequency

Unit: Mean ± standard deviation

*p-value was calculated by repeated measures ANOVA

## Discussion

FIS is known to have the characteristics of a low frequency noise. Low frequency noise (20–200 Hz) is common background noise in a city environment, typically generated by automobiles, airplanes, industry machinery, air conditioners, FIS, etc. and has a lower effect for noise reduction through the wearing of a hearing protection device or a wall, compared to other types of noise. A low frequency noise of high intensity causes injury to the respiratory system and otalgia, but the effect of a low frequency noise of low intensity has not been clearly demonstrated. Some studies have reported greater effects than other types of noise of the same level of intensity on annoyance, decreased linguistic comprehension, sleep disturbance, inability to focus, etc. [23, 24–26]. To date, little research had been conducted, specifically on FIS compared to more general types of noise. Accordingly, in the present study we aimed to investigate the effect of FIS on health and particularly on stress responses by using subjective and objective indices.

In the present study, SPL was higher in conditions without soundproof mattress than with soundproof mattress. In addition, the variation of SPL among different types of FIS without soundproof mattress was larger than used with them. The noise reduction effect of the soundproof mattress for tapping machine showed, approximately 5 dBA.

The frequency analysis on FIS showed that the conditions wherein the impact ball was used alone or in combination tended to have a relatively high SPL level in a low frequency band of 31.5–125 Hz, and the variation was even greater with a soundproof mattress (approximately a difference of 10 dBA). It seemed that the variation was greater with a soundproof mattress, because a soundproof mattress (M) had a noise reduction effect for the tapping machine to some extent, whereas it had little noise reduction effect for the impact ball. In the conditions wherein the tapping machine was used without a soundproof mattress, SPL was approximately 10 dBA higher in a high frequency band of 2000–4000 Hz, whereas there was no such difference when a soundproof mattress was used. In summary, the tapping machine used to create light weight impact sound tends to emit sound in a high frequency band and the sound it generates is somewhat reduced by a soundproof mattress, whereas the impact ball tends to emit sound in a low frequency band and the sound it generates is hardly reduced by a soundproof mattress. It is consistent with a previous finding that a floor or a wall had a noise reduction effect for a high frequency noise, but not for a low frequency noise [23]. Most FIS (over 80%) are generated by children walking or running on the floor above, showing characteristics similar to those of the impact ball [27], and our finding that the sound generated by the impact ball was not reduced by a soundproof mattress suggests that FIS is likely to continue to be an issue. However, we did not observe a strong impulse of the bang machine in a low frequency band under 63 Hz, which was pointed out by a previous study [4] as a disadvantage. This suggests that additional observations should be considered in follow-up research.

According to the different FIS types on the subjective perception of annoyance, the tapping machine (T) alone and combination with another sound source (BI, IT) were perceived as an unpleasant sound and disturbing sound.

The results of the English listening test were also consistent with the findings on subjective perceptions of annoyance and displeasure. The score was lowest in the condition wherein sound was created by the combination of the bang machine and the tapping machine (BT), followed by the tapping machine alone (T) and the combination of the tapping machine and the impact ball (TI). Moreover, the score was lower in the conditions wherein the tapping machine was used alone or in combination without a soundproof mattress. The finding suggests that sound generated by the tapping machine, especially without a soundproof mattress, is not only perceived as unpleasant but also hinders task performance. In other words, it suggests that people feel more displeased and do not perform a task well in the presence of high frequency light weight impact sound compared to low frequency heavy weight impact sound. This finding is contradictory to previous findings. A survey study conducted in 2005 with approximately 1000 residents of communal housing units in the Seoul-Gyeonggi region reported that 54–63% of respondents were uncomfortable with heavy weight impact sound, and 46–50% were uncomfortable with light weight impact sound, suggesting that heavy weight impact sound is more likely to induce discomfort [28]. Another study conducted with 32 Swedish subjects reported that they were more annoyed with a low frequency noise of approximately 40 dBA than with a flat frequency noise while performing a task [26]. The finding in the present study may be contrary to those previous findings. The finding in this study may be contrary to those previous findings. We speculate this possibility because of difference in SPL and/or in the sound occurrence rate. The sound generated by the tapping machine without a soundproof mattress had 3.9–7.3 dBA SPL higher than other sound sources, and the sound occurrence rate of the tapping machine was 10 times per second, which was the reason of different to other results. In previous studies, the subjective responses like annoyance, concentration disturbance were increased when sound levels and exposure duration increased [5–10].

However, SPL, subjective perceptions and English score were not completely congruent with one another. While SPL was the highest for the combination of the tapping machine and the impact ball (TI), followed by the combination of the bang machine and the tapping machine (BT), and the tapping machine (T) alone, both annoyance and reduction of English score were highest for the combination of the bang machine and the tapping machine (BT), followed by the tapping machine (T) and the combination of the tapping machine and the impact ball (TI). Thus, annoyance and English score were not completely explained simply by SPL, and we believe SPL and the FIS type interact with each other in a complex manner. To investigate unique effects of different FIS types, the effect of SPL should be minimized in future research. Additionally, the sound occurrence rate was the highest for the tapping machine (10 times per second) and the lowest for the bang machine (once per 1.7 ± 0.2 s). Differences in the occurrence rate could have affected the effects of different floor sound sources, but occurrence rate can also be interpreted as a unique feature of each type of FIS.

Generally, cortisol is a hormone secreted to generate energy in a threatening situation, that is, when we are under stress, the stress stimulates the hypothalamus-pituitary-adrenal axis (HPA axis) which induces the adrenal cortex to secrete the hormone. The cortisol level follows the circadian rhythm wherein it is the highest when we get up in the morning and the lowest at midnight. Cortisol levels in saliva and urine accurately reflect free, physiologically active cortisol in circulation moreover, getting the specimen of saliva and urine cortisol is appropriate to avoid the stress of intravenous sampling [29, 30]. Most previous studies have shown that exposure to noise beyond a certain level elevates the cortisol level, although there are a few studies that did not observe a correlation between noise and cortisol [11]. Different studies, however, have reported different threshold levels (e.g., over 60 dB [12], over 70 dB [13], over 80 dB [14], and over 90 dB [15]). Additionally, the cortisol level was higher before noon in some studies [12] and in the evening in other studies [13, 14].

In this study, the urinary cortisol level showed a decreasing trend after exposure to FIS but the change was statistically not significant. This finding can be contrary to the previous studies that showed elevation of cortisol level to increased noise [11–17]. However, in this study setting, we cannot exclude the influence of circadian rhythm to cortisol level. In previous studies, the diurnal variation in the cortisol levels from morning to evening showed a dereasing trend and cortisol levels were different at same time on the other days [13, 14, 17].

The salivary cortisol level significantly changed across the FIS types (*p* = 0.003), but it showed an overall decreasing trend. The finding is contrary to a previous finding that the cortisol level elevated following exposure to a low frequency noise [26], suggesting a potential involvement of the changes in cortisol levels according to circadian rhythm. Further studies must include adjustments based on fluctuations in cortisol levels according to circadian rhythm. However, one notable finding in the present study is that although the salivary cortisol level followed an overall decreasing trend, it was rather elevated when participants were first exposed to FIS (simple sound with a soundproof mattress) and when the soundproof mattress was removed for the first time (simple sound without a soundproof mattress). This can be interpreted as the salivary cortisol level being affected by exposure to FIS and the removal of the soundproof mattress.

The autonomic nerve system primarily controls heart rate, which constantly changes with irregularity depending on the internal/external environment, with this irregularity more clearly observed in a healthy person. HRV decreases if the sympathetic nerve system is activated by stress, and based on this principle, the HRV test is used as a stress test and as an assessment of balance in the autonomic nerve system [31]. SDNN and TP are mainly indicative of the overall activity of the autonomic nervous system, while HF and nHF are mainly indicative of parasympathetic activities; LF and nLF are mainly indicative of sympathetic activities, and finally, the LF/HF ratio is indicative of the balance in the autonomic nervous system [32]. Most of the studies analyzing the relationship between noise and HRV have shown that in general, the sympathetic nerve system is activated with an increase in noise, although there are variations according to the noise level and the HRV indices. In a 2007 study conducted in Germany with 110 people, SDNN increased under 65 dB while it decreased over 65 dB [18], and in a study conducted in Taiwan with 16 people, LF and the LF/HF ratio significantly increased over 50 dB [19]. In yet another study, which investigated changes in HRV according to noise type (background noise, traffic noise, speech noise and complex noise), LF and LF/HF ratio decreased during exposure to speech noise, unlike other noise types. The finding indicates the activation of the parasympathetic nerve system, suggesting the possibility that speech noise is perceived as a rather comfortable sound. In that study, the noise level was 45 dBA [20].

In the present study, significant changes were observed in SDNN (*p* = 0.015), TP (*p* = 0.004), LF (*p* = 0.002), and HF (*p* = 0.011) across the FIS types, which would suggest that HRV may vary depending on the FIS type. However, the possibility of HRV changes occurring with time cannot be excluded, and therefore, HRV change purely due to the passage of time should be minimized in future research. As an additional finding of the study, a difference was observed when HRV was analyzed according to the presence or absence of a soundproof mattress, suggesting that a soundproof mattress was effective. With a soundproof mattress, HR (*p* = 0.025), SDNN (*p* = 0.013), TP (*p* = 0.014), LF (*p* = 0.030), and HF (*p* = 0.013) showed a significant difference, whereas without a soundproof mattress, only HR (*p* < 0.001) showed a significant difference. Thus, we observed a significant change in more indices with a soundproof mattress, which can be interpreted as a different response of the autonomic nerve system to the variations of FIS when a soundproof mattress was removed.

In this study, we assessed the responses to FIS by subjective feeling, task performance ability, cortisol, HRV test. The significance of the present study is the first pilot study investigating the effect of different types of FIS on the human. We simulated diverse FIS conditions using three standard floor impact sources as well as a soundproof mattress. And we collected a wide array of subjective and objective responses, including subjective feeling, task performance, cortisol and HRV according to different FIS types to evaluate the effect of different types of FIS on the human. We expected that the methods used in the present study may provide useful information in designing future research on the effect of FIS on the human and developing a new soundproof device for FIS and establishing polices for FIS standards.

There are a few limitations for this study. First, the sample size was small (*n* = 15). Second, the exposure duration for each type of FIS was too short, and participants were not given sufficient time to rest between exposures. Third, SPL of each type of FIS was not uniform. Fourth, the influence of changes in cortisol levels according to the circadian rhythm on study findings was not minimized. Fifth, the possibility of HRV changes with time was not controlled. Sixth, the floor of university building is different from apartment, so the result may be different in apartment. Finally, the unexpected noise, which is the most stressful situation about FIS in daily living, was not fully reflected in this study because of experimental situation such as informed consent and time limitation, although the difference of FIS could be contributed to unexpected. Hence, future research should be conducted with a larger sample size over a longer experimental duration to investigate unique effects of different FIS types in further detail. Further experiments should be designed to control for a natural change with time, such as circadian rhythm and similar to daily living, such as floor thickness, buffer materials and unexpected sound. Additionally, it would be of interest to examine different intensity levels of floor impact sources and different rates of sound occurrence.

## Conclusions

In conclusion, this was the first pilot study to assess the responses of subjective feeling, task performance ability, cortisol and HRV for the different types of FIS. The responses were different according to the different types of FIS. Tapping machine without mattress induced more adverse effect in subjective feeling and performance ability. The changes of cortisol and HRV were significant. However the interpretation was partially unclear because of small sample size and confounding factors, for example, circadian rhythm of cortisol, sound level difference, sound occurrence rate and short exposure duration. And the experimental situation was different from daily living situations, such as floor thickness, buffer materials and unexpected sound. Based on this pilot study, future study is needed to control confounding factors more strictly, and to design more similar to daily living.

## Abbreviations

B: Bang machine
HF: High frequency
I: Impact ball
LF: Low frequency
M: Soundproof mattress
nHF: normalized high frequency
nLF: normalized low frequency
RMSSD: Square root of the mean of the sum of the square of differences between adjacent NN intervals
SDNN: Standard deviation of normal to normal
T: Tapping machine
TP: Total power
